# Monthly Summary

**Published:** 1877-02

**Authors:** 


### MONTHLY SUMMARY.

*Valuable Mouth Washes.*—A Wash To Harden the Gums.—Take ½ pint Jamacia spirits, ½ teaspoonful each of powdered alum and saltpetre, pulverized, and 1 oz. of pulverized myrrh ; mix.

Sozodont very much in use.—Take salts of tartar, "carbonate of potassa," ½ oz, ; honey, 4 ozs. ; alcohol, 2 ozs. ; water, 10 ozs. ; oil wintergreen and oil of rose, sufficient to flavor.

For Unhealthy Gums.—A very common thing among people are unhealthy gums, and where such is the case a lotion made from the following receipt will be found valuable in restoring them to a healthy condition :

Carbolic acid, 20 drops ; spirits of wine, 2 drachms ; *distilled water* 6 *ounces.* By first using a soft tooth brush with water, after which pour on a second tooth brush, slightly damped, a little of the above lotion. After using this for a short time the gums become less tender, and the impurity of the breath, which is commonly caused by bad teeth will be removed.—*Dental Sci.*

---

*Method of Repairing Registering Thermometers.*—The first method is to apply gentle heat to the bottom of the thermometer until the mercury rises to an expansion at the top of the glass. Permitting a little to enter this expansion, a sharp quick blow on the top breaks the column in a number of places. Shaking all down into the lower bulb, a little remains at the top to form the needle. This is forced out by heat, and partially shaken down, when the instrument is again serviceable.

In the second method, the bulb is cooled till a small space is visible above the mercury. Then the instrument is quickly inverted and held perpendicularly between the thumb and finger. In this position a sharp blow is struck, ramrod fashion, upon a thick, flat, rubber eraser, laid upon a table. A minute portion of the mercury will so be thrown past the empty space into the tube, and may serve as an index.—*Boston Med. Journal.*

---

*A Fine Tooth Paste.*—Take red coral, 3 ounces ; cuttle fish bone, 1 ounce ; disulphate of quinine, ½ drachm ; mix. Trituate to a very fine powder, add honey "white,' 4 ounces, and a few drops ottar of roses, or neroli, dissolved in rectified spirits, 3 fluid drachms, and beat the whole to a paste. A little powdered myrrh, 1 to 3 drachms, is sometimes added.—*Dental Science.*

---

*The Toxic Properties of Glycerin.*—1. Glycerin, chemically pure, causes in the dog, in twenty-four hours after being introduced under the skin, deadly effects when the dose is in the proportion of one drachm to a pound of the weight of the dog. 2. The toxic effects (acute glycerism) are comparable within certain limits to those of acute alcoholism. 3. The microscopic lesions in glycerism are analogous to those of alcoholism, which tends to induce the belief that the toxic effects of these substances are almost the same. 4. From a therapeutic point of view, there may, therefore be some danger in introducing glycerin into the economy in too large quantities.—[*Bull Gen. de Therap.*

---

*The Function of the Uvula.*—Dr. Horace Dobell in the *British Medical Journal* says:

"Looking to-day, in to the pharynx of a patient suffering from a severe nasal catarrh, I saw the watery secretions from the back of the nose pouring down in a continuous stream from the tip of the uvula into the dorsum of the tongue. It was evident that they were collected to this point from all the surrounding parts, and that the uvula acted as a conduit to bring them to the front of the epiglottis, whence they might be safely carried down the throat by repeated acts of deglutition; whereas, had it not been for the uvula, they would be liable to drip behind the epiglottis, and thus cause constant discomfort by getting into the larynx. This very simple but important function of the uvula has not, so far as I am aware, been noticed before, notwithstanding all that has been written about this odd little organ."

---

*Influence of Anæsthetics upon the Sexual Impressions of Females.*—A writer in the *Lyon Medical Clinic*, says it is a well established fact that, occasionally, under the influence of ether or chloroform, an excitation of the sexual organs is produced, and a feeling is excited in the mind by this sensation which may make a woman believe that she has been subjected to violence. In illustration of this statement, the writer says that, during delivery, he placed the woman under chloroform. The sexual sensations of the woman were so vivid that she accused him of having violated her. Yet her husband and a dozen women had been present the entire time of the delivery. Other illustrations are given, from which the wise moral is deduced, that " physicians should never administer ether or chloroform except in the presence of witnesses."—*Detroit Review.*

---

*Is Alcohol Food.*—Alcohol may, *therapeutically,* subserve the purposes of food under certain *abnormal* circumstances of the system, either by *retarding retrograde metamorphosis,* and permitting recuperation to become established, or, given in small doses, and acting as a moderate irritant or stimulant, by increasing the digestion and assimilation of food, but alcohol is not itself a direct food as we understand the definition of the term.

If there be a system of trophic nerves presiding over the nutrition of the body, as maintained by Samuel, we may safely admit, from the facts of experience, that alcohol possesses the power stimulating this system of nerves, or the cerebro spinal nerves, which preside over assimilation, whether there be or be not a special system of exclusively trophic nerves, so as primarily to increase the activity of nutritive appropriation. This property of alcohol will make lean men who take it fat, if they feed well at the same time, and do not too long continue the alcohol, to the point of nervous enervation, and, provided, that the nutrient and the alcohol are taken into the system at about the same time, so that the trophic, or nutrient nerves, while being acted upon by alcohol will not expend their increased activity fruitlessly to the system.—*Dr. Hughes, in Clinical Record*

---

*Cremation as a Science.*—Despite the recent effort to popularize cremation by the final disposal of a famous corpse, the public are as much, if not more inclined to old-fashioned burial than ever. In fact, as the result of the recent exhibition in Pennsylvania, cremation as a science has lost ground. The furnace, as exhibited on the occasion to which we refer, was a very complete one of its kind, and its construction was based upon strictly scientific principles—in fact, it accomplished everything anticipated. So far as the corpse was concerned, everything went off satisfactorily, but whether other corpses are likely to follow the example is a question. It is a pity, however, that such a perfect furnace should not be turned to some more useful purpose. —*Med. Recorder.*

